# Measuring Patients’ Anxiety Before and After MRI Examination in Taif City

**DOI:** 10.7759/cureus.103047

**Published:** 2026-02-05

**Authors:** Sultan Alamri

**Affiliations:** 1 Department of Radiological Sciences, College of Applied Medical Sciences, Taif University, Taif, SAU

**Keywords:** awareness, claustrophobia, mri safety, patient anxiety, quessionnaire

## Abstract

MRI plays a major role in diagnosing a wide range of pathological conditions, yet many patients experience anxiety before and during the scan. This study examined the degree of patient anxiety before and after MRI examinations in Taif City, as well as the factors contributing to heightened anxiety. A cross-sectional survey of 184 patients was conducted, and the results showed that anxiety was higher prior to the examination, influenced by claustrophobia, noise levels, and insufficient pre-scan information. Women and older adults reported significantly higher anxiety levels. These findings highlight the importance of improved patient communication, education, and anxiety-management measures to enhance scan tolerance and avoid premature termination.

## Introduction

MRI is widely recognized as one of the most advanced diagnostic technologies for evaluating soft tissues, musculoskeletal structures, and neurological abnormalities without the use of ionizing radiation, distinguishing it from CT and X-ray imaging techniques [1]. Despite these benefits, MRI examinations are known to induce varying levels of anxiety among patients due to factors such as loud gradient noise, the enclosed structure of the bore, prolonged scan duration, and unfamiliarity with the environment [2].

Previous studies report that approximately 30% of patients experience moderate to high anxiety before an MRI examination, and around 5% prematurely terminate the scan because of fear or claustrophobia [3-7]. Increased anxiety is strongly associated with patient movement during scanning, resulting in motion artifacts and degraded image quality. Claustrophobia accounts for a considerable portion of failed MRI studies [7,8]. Additionally, the intense acoustic noise generated by MRI scanners has been found to elevate stress levels in sensitive patients [9,10].

Since patient anxiety may hinder scan performance, prolong examination time, or necessitate sedation, it is critical to identify specific causes of MRI-related anxiety and evaluate patient experiences. This study aims to measure patient anxiety before and after MRI scans in Taif City, while examining how factors such as age, gender, claustrophobia, noise perception, and staff communication impact anxiety levels.

## Materials and methods

A descriptive cross-sectional study was conducted between October 2021 and May 2022 in Taif City, Saudi Arabia. Ethical approval was obtained from the Taif Research and Studies Department. A total of 184 adult patients (≥18 years) who were scheduled for an MRI examination during the study period, clinically stable, and able to communicate in Arabic were recruited. Data collection was carried out at King Faisal Medical Complex (KFMC) and King Abdulaziz Hospital (KAASH) using a structured questionnaire that was translated into Arabic and administered verbally before and after the MRI examinations. Patients were excluded if they were younger than 18 years, clinically unstable, unable to provide reliable responses due to cognitive impairment, or unable to communicate in Arabic.

The pre-MRI anxiety assessment questionnaire consisted of seven items assessing previous MRI experience (one item), claustrophobia (one item), awareness of MRI noise (two items), and anxiety level using a numerical Likert scale ranging from 0 to 10 (one item). Two additional items were included to collect demographic information, specifically participants’ age and gender (Appendix 1). The post-MRI anxiety assessment questionnaire consisted of six items evaluating participants’ MRI experience following the examination (Appendix 2).

For data analysis, responses were categorized as follows. Age was dichotomized into two groups: participants younger than 40 years and those aged 40 years or older. For questionnaire items with four response options (never, barely, to some extent, and more often), responses were grouped as “No” (never and barely) and “Yes” (to some extent and more often). For Likert-scale anxiety items, responses were classified as “No” if the score was less than 5 and “Yes” if the score was 5 or higher.

**Figure 1:**
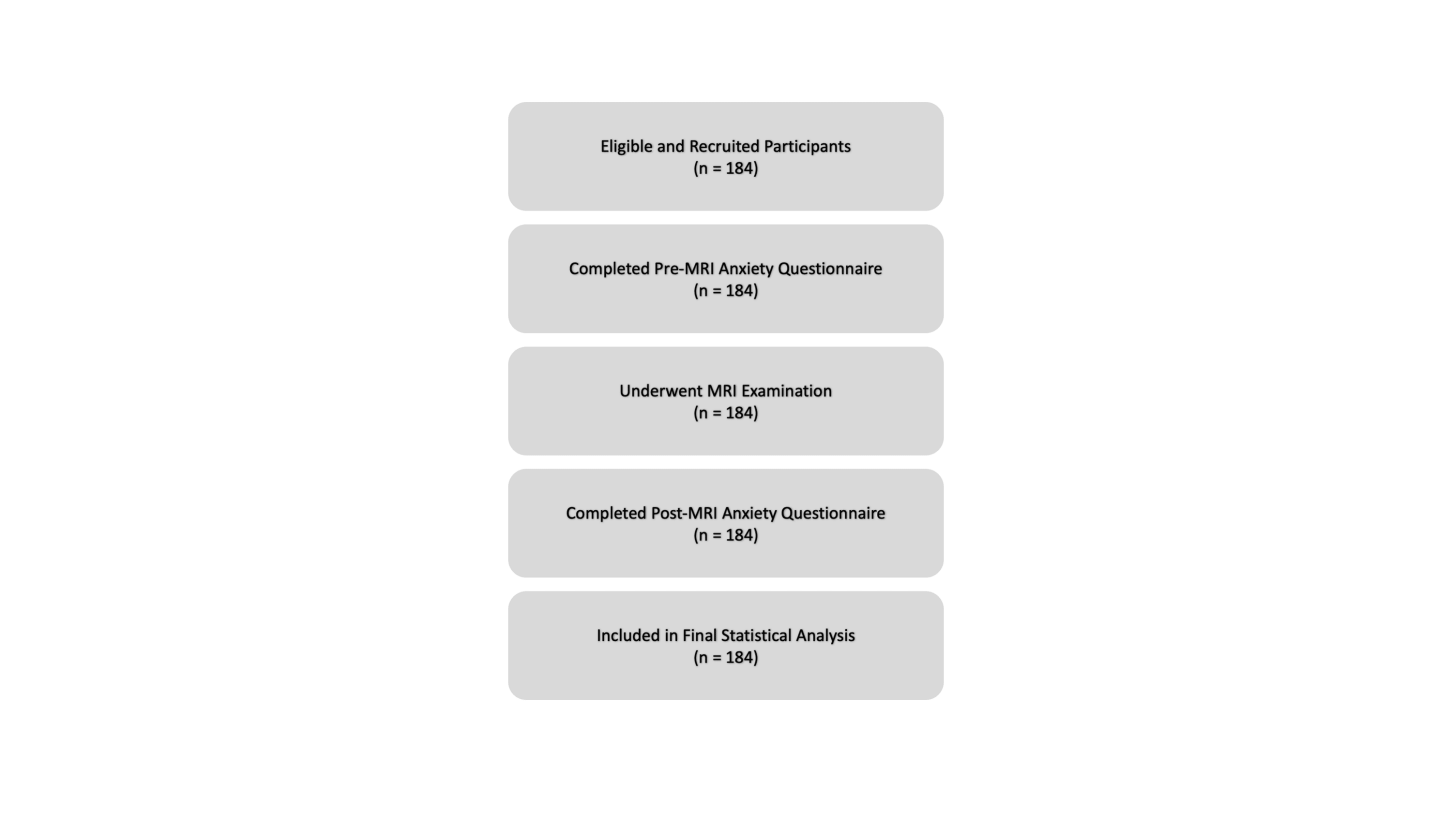
STROBE flow diagram of participant recruitment, exclusions, and inclusion in the final analysis. STROBE: Strengthening the Reporting of Observational Studies in Epidemiology.

Statistical analysis was performed using SPSS software. Chi-square tests were applied to examine associations between anxiety levels and demographic or procedural variables. McNemar’s test was used to assess changes between pre- and post-MRI responses. The participant selection process is illustrated in the STROBE flowchart (Figure 1). Statistical significance was defined as a p-value < 0.05.

## Results

A total of 184 participants completed the survey, consisting of 41% (n = 76) males and 59% (n = 108) females. The cohort included 56% (n = 103) under age 40 and 44% (n = 81) aged 40 and above. The distribution across hospitals was 63% (n = 116) at KAASH and 37% (n = 68) at KFMC.

A total of 184 patients completed the Pre- and Post-MRI anxiety questionnaire, allowing for a comparative assessment of changes in awareness, perceived knowledge, and anxiety levels before and after the examination (Table 1). The analysis focused on identifying the factors that significantly influenced MRI-related worry (Q3: How anxious were you during the MRI scan?) in the Post-MRI anxiety questionnaire compared with Q2 (How anxious were you before undergoing an MRI?) in the Pre-MRI anxiety questionnaire, as well as evaluating the role of demographic variables, specifically gender and age, in shaping patients’ responses to the individual questionnaire items.

**Table 1:** Pre- and post-MRI patient responses.

Question	No, n (%)	Yes, n (%)
Pre-examination		
Have you ever had an MRI scan?	81 (44%)	103 (56%)
Are you worried?	135 (73%)	49 (27%)
Are you afraid of closed spaces?	131 (71%)	53 (29%)
Are you aware of the noise produced by the MRI machine?	56 (30%)	128 (70%)
Are sounds a source of anxiety for you?	129 (70%)	55 (30%)
Post-examination		
Have you received information explaining the examination?	28 (15%)	156 (85%)
Did you find this information useful?	34 (18%)	150 (82%)
Were you worried during the scan?	132 (72%)	52 (28%)
Are you satisfied with the exam?	19 (10%)	165 (90%)
Was the MRI acoustic noise a source of anxiety?	124 (67%)	60 (33%)
Did the staff reassure you during the scan?	16 (9%)	168 (91%)

Overview of response patterns

Participants demonstrated varying degrees of awareness, prior experience, and anxiety associated with MRI examinations. Comparisons of Pre- and Post-MRI anxiety questionnaire responses revealed a general trend of reduced worry and improved understanding following the procedure. This indicates that patient education, staff communication, and the actual experience of the MRI process likely contribute to reduced anxiety levels.

However, the extent of worry and the nature of awareness varied across gender and age groups, prompting a more detailed subgroup analysis.

Gender-based analysis

Gender differences were observed across multiple questionnaire items, both before and after the MRI examination (Table 2). Female participants consistently exhibited higher levels of concern, fear, or uncertainty across several pre-examination questions.

**Table 2:** Number of responses by gender.

Questions	No (%) Female	No (%) Male	Yes (%) Female	Yes (%) Male
Pre-examination				
Have you ever had an MRI scan?	40 (37%)	41 (54%)	68 (66%)	35 (34%)
Are you worried?	68 (63%)	67 (88%)	40 (37%)	9 (12%)
Are you afraid of closed spaces?	67 (62%)	64 (84%)	41 (38%)	12 (16%)
Are you aware of the noise produced by the MRI machine?	30 (28%)	26 (34%)	78 (72%)	50 (66%)
Are sounds a source of anxiety for you?	65 (60%)	64 (84%)	43 (40%)	12 (16%)
Post-examination				
Have you received information explaining the examination?	16 (15%)	12 (16%)	92 (85%)	64 (84%)
Did you find this information useful?	20 (19%)	14 (18%)	88 (81%)	62 (82%)
Were you worried during the scan?	75 (69%)	57 (75%)	33 (31%)	19 (25%)
Are you satisfied with the exam?	10 (9%)	9 (11%)	98 (90%)	67 (88%)
Was the MRI acoustic noise a source of anxiety?	68 (63%)	56 (74%)	40 (37%)	20 (26%)
Did the staff reassure you during the scan?	9 (8%)	7 (9%)	99 (92%)	69 (91%)

Pre-examination Findings

Out of the total 184 participants, 103 (56%) reported having undergone MRI procedures. Of those, 66% (n = 68) were females and 34% (n = 35) were males. Interestingly, only 27% (n = 49) of participants reported feeling worried prior to the examination. Further analysis revealed a clear gender difference in pre-examination anxiety levels, with female participants demonstrating higher anxiety compared to males. Specifically, 37% (n = 40) of females reported pre-MRI anxiety compared with only 12% (n = 9) of males (p = 0.000).

Moreover, 29% (n = 53) of all participants reported claustrophobic tendencies. A marked gender difference was observed, with 38% (n = 41) of female participants reporting anxiety in enclosed spaces compared with only 16% (n = 12) of male participants (p = 0.001).

Finally, among the 184 participants, heightened sensitivity to loud noises was reported by 30% (n = 55) of the total sample. This sensitivity was more prevalent among female participants, with 40% (n = 43) of female participants reporting noise sensitivity compared with only 16% (n = 12) of male participants (p = 0.000).

These results confirm that, before the examination, female patients experience significantly higher psychological and sensory stress, which should be considered in patient preparation and communication strategies.

Post-examination Findings

Following the MRI procedures, 28% (n = 52) of the total participants reported anxiety. In contrast to the pre-examination results, gender differences became less pronounced, with 31% (n = 33) of female participants reporting anxiety following the procedure compared with 25% (n = 19) of male participants. This suggests that the MRI experience helped reduce initial pre-examination concern.

In addition, overall satisfaction with the MRI examination was high among participants, with 90% (n = 98) of female participants and 88% (n = 67) of male participants reporting satisfaction. Additionally, effective staff communication and reassurance during the MRI procedures were reported by 91% (n = 168) of participants. Among these, 92% (n = 99) were female and 91% (n = 69) were male.

Age-based analysis

Participants were divided into two groups: below 40 years (n = 102) and 40 years or older (n = 82) (Table 3). Overall, age-related differences in pre- and post-examination responses were minimal and did not indicate substantial variation between age groups.

**Table 3:** Number of responses by age.

Questions	No (<40), n (%)	No (≥40), n (%)	Yes (<40), n (%)	Yes (≥40), n (%)
Pre-examination				
Have you ever had an MRI scan?	57 (56%)	24 (29%)	45 (44%)	58 (71%)
Are you worried?	76 (75%)	59 (72%)	26 (25%)	23 (28%)
Are you afraid of closed spaces?	72 (71%)	59 (72%)	30 (29%)	23 (28%)
Are you aware of the noise produced by the MRI machine?	38 (37%)	18 (22%)	64 (63%)	64 (78%)
Are sounds a source of anxiety for you?	76 (75%)	53 (65%)	26 (25%)	29 (35%)
Post-examination				
Have you received information explaining the examination?	12 (12%)	16 (20%)	90 (88%)	66 (80%)
Did you find this information useful?	17 (17%)	17 (21%)	85 (83%)	65 (79%)
Were you worried during the scan?	75 (74%)	57 (70%)	27 (26%)	25 (30%)
Are you satisfied with the exam?	11 (11%)	8 (10%)	91 (89%)	74 (90%)
Was the MRI acoustic noise a source of anxiety?	71 (70%)	53 (65%)	31 (30%)	29 (35%)
Did the staff reassure you during the scan?	9 (9%)	7 (9%)	93 (91%)	75 (91%)

Pre-examination Findings

Older adults (≥40 years) showed higher familiarity with MRI scans, with 71% (n = 58) reporting prior MRI experience compared with 44% (n = 45) among patients younger than 40 years, suggesting more accumulated exposure due to age-related medical conditions requiring imaging. This difference was statistically significant (p = 0.000), indicating older adults are more likely to have previously undergone MRI, which aligns with clinical expectations.

Moreover, older adults demonstrated slightly higher pre-examination anxiety, with 28% (n = 23) reporting increased worry compared with 25% (n = 26) among younger patients. Although older participants showed slightly higher anxiety levels, this did not reach statistical significance (p > 0.05). Nonetheless, the direction of the results suggests that older adults may still perceive MRI as a more stressful experience.

Post-examination Findings

After completing the MRI examination, most participants felt that they had received clear and adequate information about the procedure. Although this was reported slightly more often by participants under 40 years of age (88%, n = 90) compared with those aged 40 years and older (80%, n = 66), this difference was not statistically significant (p > 0.05). Additionally, a proportion of participants continued to experience anxiety during the scan, with slightly higher levels reported among older participants (30%, n = 25) compared with younger individuals (26%, n = 27); however, this difference did not reach statistical significance (p > 0.05). Similarly, MRI-related acoustic noise remained a source of discomfort for some participants after the examination, affecting 30% (n = 31) of those aged <40 years and 35% (n = 29) of those aged ≥40 years, with no statistically significant difference between the groups (p > 0.05).

Despite these concerns, overall satisfaction with the MRI experience remained consistently high across both age groups: 89% (n = 91) in participants aged <40 years and 90% (n = 74) in those aged ≥40 years. Notably, more than 90% of participants in both age categories reported feeling reassured by staff during the scan, underscoring the central role of effective communication in promoting a positive patient experience and helping to alleviate procedural anxiety.

## Discussion

The findings of this study provide a comprehensive overview of patient anxiety associated with MRI examinations in Taif City and reveal clear differences between the pre- and post-examination phases. Overall, anxiety was more prominent before the MRI scan and decreased after completion of the procedure, supporting previous evidence that anticipatory fear plays a major role in MRI-related distress [1,2,6,7,10,11]. This reduction following the scan suggests that the actual MRI experience, when supported by appropriate staff interaction and communication, can positively reshape patient perceptions [12].

Consistent with previous studies, pre-examination anxiety in this cohort was largely driven by claustrophobia, fear of confinement, and sensitivity to acoustic noise [9]. These factors have been repeatedly identified as leading causes of MRI intolerance, scan interruption, and degraded image quality due to motion artefacts [3]. In the present study, female participants reported significantly higher levels of pre-examination anxiety, claustrophobic tendencies, and noise sensitivity than male participants. This aligns with earlier research demonstrating greater MRI-related anxiety and sensory sensitivity among women [7,13,14], potentially reflecting differences in emotional processing, stress perception, or prior healthcare experiences.

Importantly, post-examination findings demonstrated a clear reduction in anxiety across both genders. While some participants continued to report discomfort during the scan, gender differences became less pronounced after the examination. Similar reductions in anxiety following MRI exposure have been reported in previous studies, suggesting that familiarity with the procedure and real-time reassurance can effectively counteract pre-scan fears [6,12,15,16]. The high satisfaction rates observed among both female and male participants further indicate that pre-examination anxiety does not necessarily translate into a negative overall MRI experience.

Age-related differences were comparatively modest across both pre- and post-examination phases. Older participants were significantly more likely to report previous MRI experience, which is consistent with greater healthcare utilization and cumulative exposure to diagnostic imaging with advancing age [13]. However, differences in anxiety levels, noise-related discomfort, and post-examination satisfaction between age groups were small and largely not statistically significant. This suggests that, when appropriate communication and support are provided, age alone does not substantially influence the patient’s overall MRI experience. This is supported by a recent study showing that age is not a significant determinant of anxiety level [13].

A key strength of the present study is the consistent finding that staff reassurance was reported by more than 90% of participants across all demographic groups. This reinforces the growing body of evidence emphasizing the pivotal role of radiographers and MRI technologists in anxiety management [4,5,11]. Effective communication, including clear explanations, empathy, and continuous reassurance during the scan, has been shown to significantly reduce patient anxiety, improve cooperation, and enhance scan success rates [6,12,15,16].

From a clinical perspective, these findings highlight the pre-examination period as a critical window for targeted intervention. Addressing concerns related to noise and confined spaces may substantially reduce anticipatory anxiety and improve patient tolerance [1,2,11]. Interventions such as audiovisual aids [10,17], pre-scan educational videos [18-20], and patient-friendly MRI designs [21-23] have been shown to be effective in reducing anxiety and improving patient experience.

## Conclusions

MRI-related anxiety is common, particularly before the examination, but it is not inevitable or unchangeable. This study shows that patient concerns are often driven by anticipation rather than the procedure itself and tend to diminish once the examination is completed. Although some differences in anxiety were observed across age and gender groups, these differences were relatively small and did not substantially influence overall patient experience.

Most importantly, the findings emphasize the central role of effective communication and staff reassurance in reducing anxiety and maintaining high levels of patient satisfaction. Clear explanations, empathetic interaction, and supportive guidance during MRI examinations can significantly improve patient comfort and cooperation. Integrating these patient-centred practices into routine MRI services may enhance both patient experience and the quality of diagnostic imaging.

## Appendices

Appendix 1 

**Table 4:** Pre-examination questionnaire.

Item	Response options / scale
Sex	- Male - Female
Age	- 10-20 - 21-30 - 31-40 - ≥41
Have you had an MRI before?	- Yes - No
How anxious were you before undergoing an MRI?	0 1 2 3 4 5 6 7 8 9 10 (Not anxious ⟷ Very anxious)
Are you claustrophobic?	- Never - Barely - To some extent - Often
Are you aware of the noise produced by the MRI machine?	- Yes - No
Do loud noises cause you anxiety or stress?	- Never - Barely - To some extent - Often

Appendix 2 

**Table 5:** Post-examination questionnaire.

Item	Response options / scale
Did you receive an explanation of the examination and what to expect before the scan (either written or verbal)?	- Yes - No
If you received information about an MRI scan, did you find this information helpful?	- Never - Barely - To some extent - Often
How anxious were you during the MRI scan?	0 1 2 3 4 5 6 7 8 9 10 (Not anxious ⟷ Very anxious)
How satisfied were you with your MRI experience?	0 1 2 3 4 5 6 7 8 9 10 (Very dissatisfied ⟷ Very satisfied)
Did the sounds from the MRI device cause anxiety or stress?	- Never - Barely - To some extent - Often
If you were anxious during the examination, did the actions of the hospital staff reassure you?	- Never - Barely - To some extent - Often
